# The Impact of Postnatal Systemic Steroids on the Growth of Preterm Infants: A Multicenter Cohort Study

**DOI:** 10.3390/nu11112729

**Published:** 2019-11-11

**Authors:** Carlos Zozaya, Alejandro Avila-Alvarez, Fermín García-Muñoz Rodrigo, María L. Couce, Luis Arruza, Cristina Fernandez-Perez, Abdón Castro, María Teresa Cuesta, Beatriz Vacas, Máximo Vento, Miguel Saenz de Pipaón

**Affiliations:** 1 Division of Neonatology, Hospital for Sick Children, Toronto, ON M5G 1X8, Canada; 2 Neonatal Unit, Department of Paediatrics, Complexo Hospitalario Universitario A Coruña, 15006 A Coruña, Spain; alejandro.avila.alvarez@sergas.es; 3 Division of Neonatology, Complejo Hospitalario Universitario Insular Materno-Infantil, Las Palmas de Gran Canaria, 35016 Las Palmas, Spain; fgarciamu@gmail.com; 4 Neonatology Department, Complexo Hospitalario Universitario de Santiago de Compostela, Santiago de Compostela, 15706 A Coruña, Spain; Maria.Luz.Couce.Pico@sergas.es; 5 Division of Neonatology, Hospital Clínico San Carlos, 28040 Madrid, Spain; luisarruza@yahoo.es; 6 Division of Preventive Medicine, Hospital Clínico San Carlos, 28040 Madrid, Spain; cfernandezp@salud.madrid.org; 7 Neonatal Unit, Department of Pediatrics, Complejo Hospitalario de Navarra—Hospital Virgen del Camino, 31008 Pamplona, Navarra, Spain; abdoncq@gmail.com; 8 Neonatal Unit, Department of Paediatrics, Hospital Infanta Cristina, Parla, 28981 Madrid, Spain; teresitacr82@hotmail.com; 9 Neonatal Unit, Complejo Asistencial de Salamanca, 37007 Salamanca, Spain; madridbv@hotmail.com; 10 Neonatology Department, Hospital Universitari i Politècnic la Fe, 46026 Valencia, Spain; Maximo.Vento@uv.es; 11 Neonatology Department, Hospital Universitario La Paz, 28046 Madrid, Spain; miguel.saenz@salud.madrid.org

**Keywords:** bronchopulmonary dysplasia, preterm infant, steroids, growth

## Abstract

Postnatal steroids, often used to prevent and treat bronchopulmonary dysplasia, may influence the growth of preterm infants, although data are scarce in the literature. This is a multicenter cohort study including surviving preterm infants <32 weeks at birth (*n* = 17,621) from the Spanish Neonatal Network SEN1500 database, without major congenital malformations. Linear regression models were adjusted for postnatal steroids, respiratory severity course (invasive mechanical ventilation at 28 days), progression to moderate–severe bronchopulmonary dysplasia (O_2_ at 36 weeks), length of stay, sex, gestational age and z-scores at birth. A subgroup analysis depending on the timing of administration, ventilation status at 28 days and moderate–severe BPD diagnosis was also performed. Overall, systemic postnatal steroids were not independently associated with poorer weight gain (0.1; 95% CI: −0.05 to 0.2 g/kg/day), linear growth (0; 95% CI: −0.03 to 0.01 cm/week) or head circumference growth (−0.01; 95% CI: −0.02 to 0 cm/week). Patients who received steroids after 28 days or who were not O_2_ dependent at 36 weeks after having received steroids gained more weight (0.22; 95% CI: 0.04 to 0.4 and 0.2; 95% CI: 0.004 to 0.5 g/kg/day, respectively). Globally, systemic postnatal steroids had no significant adjusted effect on postnatal growth.

## 1. Introduction

Postnatal growth restriction is one of the most common complications of prematurity and is associated with worse neurodevelopment [1]. Postnatal growth restriction is the result of a combination of different factors, leading to insufficient nutrient delivery, absorption or accretion to match the protein-caloric requirements of a rapidly growing neonate, who faces a number of organ and systemic complications. Postnatal growth restriction may occur even when current nutritional recommendations are followed [2]. Other factors independent of nutrient delivery, sex and illness may affect postnatal growth. For instance, medical and surgical treatments can interfere with growth too. Better knowledge of the interactions between treatments, disease, metabolism, and growth may increase our understanding of the etiology of postnatal growth restriction and, eventually, lead to the design of better strategies to prevent it.

Steroids are often used in preterm infants during their admission to the Neonatal Intensive Care Unit. Steroids can be indicated to treat refractory hypotension or to facilitate extubation. More commonly, steroids are used to reduce the risk and severity of bronchopulmonary dysplasia (BPD) [3]. Courses of steroids to prevent/treat BPD are usually longer and involve higher doses than the indications mentioned above. However, steroids also have potential adverse effects, including hypertension, hyperglycemia, adrenal suppression and growth impairment, at least regarding height, in children [4,5]. Dexamethasone has growth-suppressive and catabolic effects, which may have long-term consequences on growth and organ development [6]. Indeed, former preterm infants also have a reduced growth rate during the first years of life if they had received steroids during the neonatal period [7]. However, little is known about the overall effect of postnatal steroids on intra-hospital postnatal growth beyond its acute effect on weight gain velocity during steroid treatment [8]. We aimed to evaluate and quantify the effect of postnatal steroids given to prevent or to treat BPD before discharge on the intra-hospital global growth of the very and extremely preterm infant. 

## 2. Materials and Methods 

### 2.1. Study Design and Population

This is an observational multicenter cohort study. Data have been obtained from the Spanish Neonatal Network SEN1500 database, where participating Spanish neonatal units collect data prospectively about every infant admitted with birthweight <1500 g [9]. The only inclusion criteria for analysis in this study was gestational age <32 weeks at birth. Exclusion criteria were death before first discharge, major congenital malformations and discharge before 28 days of life. The study period was from 1 January 2005 to 31 December 2015. 

Outcome variables were weight gain velocity (g/kg/day), linear growth (cm/week) and head circumference growth (cm/week) from birth to discharge. Weight gain (g/kg/day) was calculated as per the method suggested by Patel et al. [10]. Anthropometric data were obtained as per routine unit practices and collected at birth and discharge. Z-scores according to sex and gestational or postmenstrual age were obtained using either Fenton’s 2013 before 50 weeks postmenstrual age (PMA) or World Health Organization growth pattern references, after 50 weeks PMA [11,12]. Changes in weight, length and head circumference z-scores were calculated from birth to discharge as the difference between discharge and birth z-scores. BPD was defined as oxygen need at 28 days of life and classified as moderate–severe BPD if oxygen was required at 36 weeks postmenstrual age [13]. Steroid treatment was collected as a categorical dichotomic variable in the original database. Only steroids indicated for BPD prevention or treatment were considered. Short and lower-dose courses of steroids for extubation or refractory hypotension were not considered. Age at the beginning of steroid initiation was also available on the database. The indication for steroid treatment (the type of steroid, duration, route of administration and dosage) depended on the attending physicians. 

### 2.2. Statistical Analysis

Continuous variables are expressed as the mean (standard deviation) or median (interquartile range) and compared by the Student’s t-test or Mann–Whitney U test, as indicated. Qualitative variables are expressed as proportions (n/N) and percentage (%) and analyzed by the Chi-square or Fisher’s exact test, as appropriate. Patients with missing values were excluded from the analysis. All comparisons were two-tailed, and a *p*-value <0.05 was considered statistically significant. We built linear regression models adjusting by factors associated both with postnatal growth restriction and BPD: invasive mechanical ventilation at 28 days of life, length of stay, sex, gestational age, and z-scores at birth (i.e., birth weight z-score in models addressing weight gain). Ventilation at 28 days was considered as a surrogate of respiratory disease severity, and moderate–severe BPD as a surrogate of steroid ineffectiveness to avoid BPD progression. Therefore, models looking for interactions between steroids and ventilatory status at 28 days and steroids and the severity of BPD were also built, and a subsequent stratified analysis was performed. A subgroup analysis depending on the timing of administration before day 28 (aim to prevent BPD) and after day 28 (treatment of BPD) was also performed. Statistical analysis was performed with Stata 13.1 statistical software (StataCorp, Texas, USA). 

### 2.3. Ethical Issues

The local Ethics Research Committee of all participant centers approved the data collection protocol when they joined the network. Permission for data analysis was obtained from the executive committee of the Spanish Neonatal Network SEN1500. 

## 3. Results

Data on 17,621 preterm infants <32 weeks were included for analysis after 1141 patients with major congenital malformations, 3252 patients who died before discharge, and 952 patients who were discharged before 28 days of life were excluded. Only nine (1%) patients among those surviving preterm infants but discharged before day 28 had received steroids. Data about weight gain and changes in weight z-scores were obtained from 98% of the analyzed patients. Regarding linear growth and changes in length, z-score data were available in 86% of the patients. Finally, data about head growth and changes in head circumference z-score were collected from 87% of the patients. Data about postnatal steroids were available in 17,588 (99.8%) patients. In our cohort, 7.6% (1338/17,588) of the patients received postnatal systemic steroids. Median age at steroid course initial dose was 27 (19–36) days. The main epidemiological, growth and respiratory data on the analyzed patients are summarized in Table 1.

Overall, after adjustment by sex, gestational age, moderate–severe BPD, invasive mechanical ventilation at 28 days of life, length of stay and z-scores at birth, systemic postnatal steroids had no significant adjusted effect on postnatal weight gain (0.1; 95% CI: −0.05 to 0.2 g/kg/day), linear growth (0; 95% CI: −0.03 to 0.01 cm/week) or head circumference growth (−0.01; 95% CI: −0.02 to 0 cm/week). Systemic postnatal steroids were not associated with a bigger fall in weight (0.13; 95% CI: 0.08 to 0.19), length (0.01; 95% CI: −0.06 to 0.09) or head circumference (−0.05; 95% CI: −0.13 to 0.02) z-scores from birth to discharge either.

### 3.1. Subgroup Analysis: Timing of Administration

A separate analysis, adjusting by the same variables, was performed considering patients who received steroids before (preventive steroids) or after the 28 days of life (treatment). Patients who were started on postnatal systemic steroids when they were <28 days old (43.7%) were 20 (15–23) days old at the beginning of the steroid course. Systemic postnatal steroids were given after 28 days of life in the another 56.3% of the cases (median age: 37, 30–47 days). Results of this subgroup multivariable analysis are presented in Table 2, showing that those who received steroids after 28 days gained more weight. 

### 3.2. Interaction between Postnatal Steroids and Ventilatory Status at 28 Days of Age

We used invasive mechanical ventilation at 28 days of life as a surrogate for respiratory disease severity. Among patients who required invasive mechanical ventilation at 28 days of age, 44% received steroids, while only 4.5% of the patients who were not invasively ventilated at 28 days did. Head growth restriction was observed in babies who received steroids but were not invasively ventilated at 28 days of life (full results are presented in Table 3). 

### 3.3. Interaction between Postnatal Steroids and Classification as Moderate–Severe BPD (Oxygen at 36 Weeks Postmenstrual Age)

Patients who eventually developed moderate–severe BPD received steroids in 33% of the cases, whereas only 3.1% of the infants without moderate–severe BPD at 36 weeks had previously received steroids. The potential interaction between postnatal steroids and final classification of a case as moderate–severe BPD was analyzed, with a stratified multivariable analysis according to the final classification as moderate–severe BPD. Steroids were associated with greater weight gain in infants who eventually did not develop moderate–severe BPD (full results are presented in Table 4). 

## 4. Discussion

We report data showing growth outcomes during hospital admission in infants exposed to postnatal systemic corticosteroids. To our knowledge, this is the largest cohort study reporting data on weight gain but also linear and head growth during neonatal admission, concerning postnatal steroids. We found that postnatal systemic steroids had no significant adjusted effect on postnatal growth restriction. Only head growth was negatively associated with steroids in patients who were not invasively ventilated at 28 days. On the other hand, greater weight gain was observed in patients who received steroids for treatment rather than prevention (after 28 days). The same effect on weight was found in those patients who received steroids if progression to moderate–severe BPD (O_2_ at 36 weeks) was avoided.

It is known that dexamethasone acutely slows down weight gain velocity during the treatment period [8,14,15,16,17]. However, the final effect of postnatal steroids on weight beyond the end of the treatment but before hospital discharge has been seldom reported. There are even less data regarding the effect of steroids on head circumference and linear growth. A recent trial to assess the efficacy of early hydrocortisone therapy to prevent BPD did not find differences in weight and head circumference z-scores at 36 weeks between groups [18]. Another trial tested the effect of hydrocortisone initiated between days 7 and 14 in ventilated patients on mortality or BPD [19]. At 36 weeks, they found no differences in length and head circumference. Weight was greater in the hydrocortisone than in the placebo group, although it was also greater at birth. The respiratory course itself may confound the effect of steroids on growth. Tijsseling et al. compared weight gain, linear and head growth of patients who received either no steroids, dexamethasone or hydrocortisone, finding that there is an acute fall and then a delayed growth velocity affecting the three parameters in treated patients. However, no adjustment by final BPD diagnosis was made [7]. 

Data about how steroids interfere with normal growth physiology in the preterm infant are scarce. Endogenously, cortisol is a catabolic hormone, and cortisol production levels are negatively correlated with weight gain in the preterm infant [20]. Dexamethasone’s effect on metabolism has also been studied in the past. The effect of dexamethasone on growth may be mediated both by hormonal and metabolic mechanisms. Dexamethasone acutely reduces growth hormone levels, but not growth factors IGF-1, IGFBP-1 or IGFBP-3, which are more related to fetal and preterm postnatal growth. Dexamethasone also increases proteolysis in the short term, leading to a negative nitrogen balance [16,21,22,23]. Hydrocortisone is another steroid commonly used in preterm infants with or at risk for BPD. Nevertheless, the effect of hydrocortisone on preterm short-term growth, physiology and metabolism remains mainly unknown.

The severity of the respiratory course or of BPD itself is a risk factor for postnatal growth restriction [20,24]. We separately explored the interaction between steroids and ventilatory status at 28 days and with the final classification as moderate–severe BPD (O_2_ at 36 weeks). We found that the negative association between steroids and head growth is seen only on those patients who were not intubated by 28 days of life. Brownlee et al. proved that dexamethasone increased catabolism and already suggested that treating babies with milder degrees of bronchopulmonary dysplasia would have a less favorable benefit–risk ratio [23]. On the contrary, regarding the efficacy of steroids to prevent progression to moderate–severe BPD, our results may indicate that when steroids worked, the effect on growth is positive as they gained more weight.

A limitation of our study could be related to the accuracy and reliability of the results depending on the measuring instruments and the large number of researchers involved. In experimental conditions, the homogeneity in the instruments of measurement and the fact that only one or just a few researchers perform them could improve the accuracy of the results (internal validity). In multicenter studies of the type presented here, it is necessary to assume some degree of unaccounted bias mimicking the heterogeneity of measurement errors occurring in similar neonatal units worldwide, thus not affecting the generalization of results to the target patient group in clinical practice.

In addition, the analyzed data were collected over a long period, during which relevant changes in clinical practice could have taken place. For instance, a previous study comparing changes in perinatal care and outcomes in two periods (2002–2006 vs. 2007–2011) in Spain showed an increase in the proportion of very low birth weight patients who received antenatal steroids (from 80.7% to 85.0%; *p* < 0.001), and a reduction in the administration of postnatal steroids for BPD (from 6.0% to 5.3%; *p* = 0.02). This study also showed a reduction in the use of oxygen and invasive mechanical ventilation. Survival for the most immature patients (<26 weeks GA) increased from 26.6% to 36.6% (*p* < 0.001). Changes in nutritional practices were not systematically collected [25]. Data about the type of steroid, number of cycles, route of administration, dosage schedule and duration of therapy were not available in the original database. However, according to recent data from the Spanish Research Group on Bronchopulmonary Dysplasia (GEIDIS) registry, which includes 90% of the Spanish Neonatal Units, 26.5% of patients with any severity of BPD during the period 2016 and 2017 in Spain received steroids (dexamethasone 55.4%, hydrocortisone 37.2%, and others 7.4%). The total cumulative dose, mean (SD), was 1.79 (3.2) and 43.8 (35.7) mg/kg for dexamethasone and hydrocortisone, respectively. The drugs were administered mostly intravenously. Nevertheless, the effect of dexamethasone and hydrocortisone on growth may be different [7]. Regarding head growth, for instance, it is known that dexamethasone is related to poor brain growth, whereas hydrocortisone does not seem to affect brain volumes [26]. These results are relevant, since the longitudinal growth reflects the increase in lean mass and protein accretion, which is in turn an index of the growth of the organs, including the brain. Although the optimal longitudinal growth pattern is not fully known, there is recent evidence that its restriction is associated with worse long-term cognitive outcomes [27,28].

On the other hand, the big sample size and multicenter nature of our study are the stronger points of this study as we believe these results are generalizable to preterm infants treated in modern-day NICUs. 

## 5. Conclusions

In conclusion, no significant effect of postnatal steroids on overall growth was found. However, when given to patients who are not invasively ventilated at 28 days, steroids were associated with head growth impairment at hospital discharge. On the contrary, when BPD progression is avoided, steroids were associated with greater weight gain. Data about the exact pathophysiologic mechanisms involved, especially regarding hydrocortisone, are required. Once our knowledge about the underlying mechanisms improves, trials aiming to limit this adverse effect during and after steroid courses could be designed. 

## Figures and Tables

**Table 1:** Epidemiological, clinical and growth features of the study population.

Study Population (N = 17,621)			
	No steroids (N = 16,250)	Steroids (N = 1338)	*p*-value
Sex (female)	49.2% (7981/16,211)	41.5% (555/1337)	*p* < 0.001
Gestational age (weeks)	29.3 (27.9 to 30.4)	26.6 (25.4 to 27.9)	*p* < 0.001
Birthweight (g)	1140 (950 to 1320)	826 (700 to 965)	*p* < 0.001
Birthweight z-score	−0.3 (−0.8 to 0.2)	−0.3 (−0.9 to 0.3)	0.59
Small for gestational age (z-score < −1.5)	7.1% (1158/16,250)	10.5% (140/1338)	*p* < 0.001
Length at birth (cm)	37.5 (35 to 39.5)	33.5 (32 to 35.5)	*p* < 0.001
Length at birth z-score	−0.1 (−0.8 to 0.5)	−0.3 (−1 to 0.4)	*p* < 0.001
Head circumference at birth (cm)	26.5 (25 to 28)	24 (22.5 to 25)	*p* < 0.001
Head circumference at birth z-score	−0.1 (−0.8 to 0.6)	−0.2 (−0.9 to 0.3)	*p* < 0.001
Length of (first) neonatal admission (days)	55 (43 to 72.5)	101 (84 to 122)	*p* < 0.001
Bronchopulmonary dysplasia	30.4% (4831/ 15,881)	91.9% (1207/1314)	*p* < 0.001
Bronchopulmonary dysplasia (moderate–severe BPD)	13.1% (1767/13,452)	69.9% (872/1247)	*p* < 0.001
Invasive mechanical ventilation at 28 days of life	5% (759/15,272)	46.9% (602/1284)	*p* < 0.001
Weight gain (g/kg/day)	12.6 (11.1 to 14.1)	11.9 (10.4 to 13.3)	*p* < 0.001
Change in weight z-scores from birth to discharge	−1.1 (−1.7 to −0.7)	−1.7 (−2.4 to −1)	*p* < 0.001
Linear growth (cm/week)	0.9 (0.7 to 1.1)	0.9 (0.8 to 1)	0.013
Change in length z-score from birth to discharge	−1.3 (−1.2 to −0.6)	−2 (−2.9 to −1.3)	*p* < 0.001
Head growth (cm/week)	0.8 (0.7 to 0.9)	0.7 (0.6 to 0.8)	*p* < 0.001
Change in head circumference z-score from birth to discharge	−0.33 (−1 to 0.3)	−0.8 (−1.5 to −0.1)	*p* < 0.001

Data are presented as the median (interquartile range) or n/N (%), where N is the number of patients with data available. SGA = small for gestational age. BPD = bronchopulmonary dysplasia. HC = head circumference.

**Table 2:** Adjusted effect of postnatal systemic steroids on the growth parameters of preterm infants <32 weeks of gestational age according to the timing of systemic steroid administration. Regression models are adjusted by gestational age, sex, invasive mechanical ventilation at 28 days, moderate–severe BPD, length of stay, and z-score at birth for weight, length or head circumference. Changes in z-scores refer to changes in z-score from birth to discharge.

	Systemic Postnatal Steroids before 28 Days (95% CI)	Systemic Postnatal Steroids after 28 Days (95% CI)
Weight gain (g/kg/day)	−0.03 (−0.22 to 0.16)	0.22 (0.04 to 0.4) *
Changes in weight z-scores	0.1 (0.03 to 0.17) *	0.19 (0.12 to 0.26) *
Linear growth (cm/week)	−0.02 (-0.05 to 0.01)	0.0 (−0.02 to 0.03)
Changes in length z-scores	−0.07 (−0.16 to 0.03)	0.08 (−0.01 to 0.2)
Head growth (cm/week)	−0.02 (−0.04 to −0.01) *	0.0 (−0.01 to 0.02)
Changes in head z-scores	−0.1 (−0.014 to −0.011) *	0.01 (−0.08 to 0.1)

* Indicates *p* < 0.05.

**Table 3:** Adjusted effect of postnatal systemic steroids on the growth parameters of preterm infants <32 weeks of gestational age stratified by ventilatory status at 28 days of life. Regression models are adjusted by GA, sex, invasive mechanical ventilation at 28 days, moderate–severe BPD, length of stay, and z-score at birth (weight, length or head circumference). Results are adjusted associations between steroids used and growth parameters (95% CI). Changes in z-scores refer to changes in z-score from birth to discharge.

	Systemic Postnatal Steroids in Mechanically Ventilated Patients at 28 Days of Life (95% CI)	Systemic Postnatal Steroids in Non-Mechanically Ventilated Patients at 28 Days of Life (95% CI)	Interaction *p*-Value
Weight gain (g/kg/day)	0.09 (−0.1 to 0.3)	0.08 (−0.09 to 0.26)	0.15
Changes in weight z-scores	0.1 (−0.02 to 0.2)	0.13 (0.07 to 0.19)	**0.01**
Linear growth (cm/week)	−0.004 (−0.03 to 0.02)	0.01 (−0.02 to 0.03)	0.44
Changes in length z-scores	0.005 (−0.2 to 0.2)	0.025 (−0.06 to 0.11)	0.66
Head growth (cm/week)	0.004 (−0.01 to 0.02)	−0.02 (−0.03 to 0.001)	**0.03**
Changes in head z-scores	0.01 (−0.16 to 0.18)	−0.12 (−0.21 to -0.03) *	**0.01**

* Indicates *p* < 0.05 in the multivariable analysis, whereas the interaction *p*-values reflect the *p*-value of the analysis of the interaction between steroids and invasive mechanical ventilation at 28 days old. Significance is marked in bold.

**Table 4:** Adjusted effect of postnatal systemic steroids on the growth parameters of preterm infants <32 weeks of gestational age at birth stratified by moderate–severe bronchopulmonary dysplasia. Regression models are adjusted by GA, sex, invasive mechanical ventilation at 28 days, length of admission, and z-score at birth (weight, length or HC). Results are adjusted associations between steroids used and growth parameters (95% CI). Changes in z-scores refer to changes in z-score from birth to discharge.

	Systemic Postnatal Steroids (Moderate–Severe BPD (95% CI)	Systemic Postnatal Steroids (No Moderate–Severe BPD (95% CI))	Interaction *p*-Value
Weight gain (g/kg/day)	−0.05 (−0.2 to 0.1)	0.2 (950.004 to 0.5) *	0.2
Changes in weight z-scores	0.04 (−0.06 to 0.1)	0.2 (0.08 to 0.2) *	0.46
Linear growth (cm/week)	−0.01 (−0.03 to 0.02)	0.01 (−0.02 to 0.04)	0.17
Changes in length z-scores	0.02 (−0.1 to 0.1)	0.02 (−0.09 to 0.1)	0.8
Head growth (cm/week)	−0.01 (−0.02 to 0.004)	−0.01 (−0.03 to 0.005)	0.3
Changes in head z-scores	−0.03 (−0.2 to 0.09)	−0.08 (−0.2 to 0.03)	0.73

* Indicates *p* < 0.05 in the multivariable analysis, whereas the interaction *p*-values reflect the *p*-value of the analysis of the interaction between steroids and moderate–severe.

## Appendix A

Complejo Hospitalario Albacete (Andrés Martínez Gutiérrez); Hospital U. de Basurto (Alberto Pérez Legorburu); Hospital Bierzo (María Teresa Prada); Hospital Cabueñes (Rafael García Mozo); Hospital Carlos Haya (Tomás Sánchez Tamayo); Hospital General Castellón (Ramón Aguilera Olmos); Hospital Central Asturias (Belén Fernández Colomer); Hospital Clínic Barcelona (Josep Figueras Aloy); Hospital Clínico San Carlos (Araceli Corredera Sánchez); Hospital Cruces (Amaya Rodríguez Serna); Hospital U. Donostia (M. Ángel Cortajarena Altuna); Hospital Elche (Carolina Vizcaíno); Hospital General de Cataluña (Laura Castells Vilella); Hospital G. de Granollers (Israel Anquela Sanz); Hospital Germans Trias i Pujol (W. Coroleu); Hospital Getafe (Lucía Cabanillas Vilaplana); Hospital Jerez (Mª Victoria Ramos Ramos); Hospital Juan Ramón Jiménez (David Mora Navarro); Hospital Juan XXIII (Mar Albújar); Hospital León (Mª Teresa Palau Benavides); Hospital Miguel Servet (Segundo Rite Gracia); Corporació Parc Taulí (Juan Badia); Hospital San Juan de Deu (Martín Iriondo Sanz); Hospital San Pedro de Logroño (Mª Yolanda Ruiz del Prado); Hospital San Pedro de Alcántara (Mª Jesús López Cuesta); Hospital de la Santa Creu i Sant Pau (Gemma Ginovart Galiana); Hospital Severo Ochoa (María José Santos Muñoz); Hospital Txagorritxu (María Mercedes Martínez Ayucar); Hospital Universitario Arnau de Vilanova (Eduard Solé Mir); Hospital Valme de Sevilla (Josefina Márquez Fernández); Hospital Virgen de la Concha (Víctor Marugán Isabel); Hospital U. Virgen de la Macarena (Mercedes Granero Asensio); Hospital Virgen de la Salud (Ana Belén Escobar Izquierdo); Hospital U. Virgen de las Nieves (Mª Fernanda Moreno Galdó); Hospital Xeral Vigo (María Suárez Albo); Hospital Universitario de Valencia (Javier Estañ Capell); Hospital Universitario de Zaragoza (Purificación Ventura Faci); Hospital Universitario Santiago (Mª José Fernández-Seara); Hospital General de Burgos (Cristina de Frutos Martínez); Hospital General Universitario Alicante (Honorio Sánchez Zaplana); Hospital Universitario de Fuenlabrada (Laura Domingo Comeche); Hospital Universitario Gregorio Marañón (Dorotea Blanco Bravo); Hospital Universitario La Paz (Mª Dolores Elorza); Hospital Materno Infantil de Canarias (Lourdes Urquía Martí); Hospital Universitario Canarias (Pedro A. Fuster Jorge); Hospital Universitario de San Cecilio (Eduardo Narbona); Hospital Universitari i Politecnic La Fe (Isabel Izquierdo Macián); Hospital Universitario Reina Sofía (Mª Pilar Jaraba Caballero); Hospital Universitario Río Hortera (Mª Mar Montejo Vicente); Hospital Universitario Salamanca (Pilar García González); Hospital Universitario Virgen del Rocío (Carmen Macías Díaz); Hospital Universitario Dexeus (Roser Porta); Scias-Hospital Barcelona (Silvia Martínez-Nadal); Hospital Josep Trueta (Alberto Trujillo); Complejo Hospitalario Universitario de Pontevedra (Pilar A. Crespo Suárez); Hospital Universitario de Ciudad Real (Miguel Ángel Cabezas); Hospital Puerta del Mar (Antonio Segado Arenas); Hospital Universitario Marqués de Valdecilla (Isabel de las Cuevas); Hospital Quirón Málaga (Manuel Baca Cots); Hospital de la Zarzuela (M López Gómez); H. Nuestra Señora de la Candelaria (Sabina Romero); H. Madrid Torrelodones (Isabel Llana Martín); H. Puerta de Hierro (C. González Armengod); H. U. Santa Lucía de Cartagena (J.M. Lloreda García); H. Virgen del Camino de Pamplona (Concepción Goñi Orayen); H. U. Quirón Madrid (M López Azorín); Clínica Cocharán (M.D. Muro Sebastián); Complexo H. U. de A Coruña (Alejandro Ávila Álvarez); H. Quirón Sagrado Corazón de Sevilla (Elena García Victori); H.U. Fundación Jiménez Díaz (T. Carrizosa).
